# The Perception and Mimicry of Facial Movements Predict Judgments of Smile Authenticity

**DOI:** 10.1371/journal.pone.0099194

**Published:** 2014-06-11

**Authors:** Sebastian Korb, Stéphane With, Paula Niedenthal, Susanne Kaiser, Didier Grandjean

**Affiliations:** 1 Department of Psychology, University of Wisconsin-Madison, Madison, Wisconsin, United States of America; 2 Department of Psychology, University of Geneva, Geneva, Switzerland; 3 Swiss Center for Affective Sciences, University of Geneva, Geneva, Switzerland; UCLA, United States of America

## Abstract

The mechanisms through which people perceive different types of smiles and judge their authenticity remain unclear. Here, 19 different types of smiles were created based on the Facial Action Coding System (FACS), using highly controlled, dynamic avatar faces. Participants observed short videos of smiles while their facial mimicry was measured with electromyography (EMG) over four facial muscles. Smile authenticity was judged after each trial. Avatar attractiveness was judged once in response to each avatar’s neutral face. Results suggest that, in contrast to most earlier work using static pictures as stimuli, participants relied less on the Duchenne marker (the presence of crow’s feet wrinkles around the eyes) in their judgments of authenticity. Furthermore, mimicry of smiles occurred in the Zygomaticus Major, Orbicularis Oculi, and Corrugator muscles. Consistent with theories of embodied cognition, activity in these muscles predicted authenticity judgments, suggesting that facial mimicry influences the perception of smiles. However, no significant mediation effect of facial mimicry was found. Avatar attractiveness did not predict authenticity judgments or mimicry patterns.

## Introduction

The present study had two major aims. First, we were interested in investigating the facial features used by observers to judge that a smile is “authentic,” that is, that it is caused by a positive internal state. Dynamic, highly controlled stimuli were used, as recent research emphasizes the importance of dynamic features for conveying smile authenticity. Second, we recorded the activation of facial muscles in order to test the prediction, based on theories of embodied emotion, that facial mimicry supports, and possibly mediates, judgments of smile authenticity.

### What Makes a Smile Authentic?

As we go through everyday life, and interact with other people, we are exposed to a variety of facial expressions of emotion. Being able to decode, or “read” facial expressions helps us to infer others’ emotions and intentions in social interactions, and to modify our behavior accordingly. This can happen both at a conscious or unconscious level of processing. However, there is not a simple one-to-one relationship between an internal emotional state and a facial expression. This is the case because, to different degrees, adults are able to modify or suppress their facial expressions [1], [2], and to mask – consciously or unconsciously – their emotions behind a different expression altogether [3].

One of the most frequently encountered facial expressions is the smile. The smile is perhaps the most nuanced expression in that it can reflect very different underlying emotions and/or be interpreted in different ways depending on its context and on the observer’s beliefs. Imagine, for example, a sales person who has to reach a financial goal. He or she might flash a different smile depending on whether a sale has yet to be sealed, or has already been successfully accomplished. Or, two identical smiles displayed by the same salesperson might be interpreted as caused by very different states if the perceiver believes that sales people smile out of the intent to manipulate before a sale, but out of satisfied pleasure after the sale [4]. Thus, a smiler may not always feel happy, joyful, or amused – an insight already conveyed in the writings of Charles Darwin [5]. Instead, smiling may reflect affiliative intent, responsiveness to group norms, or social status [6], and people tend to make use of this knowledge when interpreting smiles.

Across the psychological and neuroscientific literature, smiles have been conceptualized and named in different ways. Smiles reflecting a positive underlying emotion have been dubbed *enjoyment*, *authentic*, *amused*, *felt*, *Duchenne*, *genuine*, *humor*, *duplay*, and *broad* smiles. Smiles not generated by an underlying positive emotion and/or used intentionally to mask negative emotions have been called *non-enjoyment*, *embarrassed*, *non-Duchenne*, *unfelt*, *false*, *social*, *masking*, and *controlled* smiles [7]. Here, we refer to these two broad categories as *true* and *false* smiles, although other terms may occasionally be used as synonyms.

Past research has attempted to identify the morphological and, to a lesser degree, dynamic features that seem to diagnose true and false smiles, both in the laboratory [7]–[15], and in more naturalistic settings [16]. The results suggest that true and false smiles are associated with different brain states during both their production [17], [18] as well as perception [19]. Moreover, the distinction between the two is relevant for predicting social behaviors. For example, people displaying true smiles are perceived as being more positive and trustworthy than those displaying false smiles [20]. Interestingly, people seem to be able to distinguish between true and false smiles even without being asked to do so, and when the pictures of smiles are presented for very brief exposures [12], [21], possibly suggesting reflexive processing and automatic feature detection during smile decoding.

Because it relies on early claims by Ekman and colleagues [8], [17], [22], most of the extant research distinguishing true and false smiles has focused on the presence of crow’s feet wrinkles around the eyes, produced by the contraction of the Orbicularis Oculi muscles (the Duchenne marker), as the defining characteristic of true smiles. However, more recent studies have shown that the Duchenne marker is sometimes present in false smiles [23], and have emphasized the relatively neglected importance of dynamic features for reliably recognizing facial expressions in general and for distinguishing between true and false smiles in particular [11], [24]. There is indeed psychological and physiological evidence suggesting that facial expressions are perceived and mimicked differently when they are presented as dynamically unfolding videos instead of still pictures depicting only the expression’s apex [25]–[27]. Dynamic facial expressions generate greater activation in brain regions associated with the processing and interpretation of social and emotional stimuli [28]. And the perception of static and dynamic aspects of facial expressions may rely upon different neural structures, as suggested by patients’ studies [29], [30].

On this basis, we suspected that the Duchenne marker plays a more nuanced role in driving judgments of the authenticity of dynamic smiles compared to its previously reported prominent role in the judgment of static smiles. Interestingly, the use of the Duchenne marker to judge smile authenticity may become acquired with practice, as it has been shown to be more common in older compared to younger children [31]. This may be due to the fact that the ability to perceive kinetic and pictorial depth information develops throughout infancy [32].

In light of the still open questions about the features that make a smile look true versus false, and of the more recent technological developments supporting the production and presentation of controlled dynamic smiles, the primary aim of the present research was to use dynamic, controlled stimuli to isolate the features that individuals rely on to diagnose the authenticity of smiles. For this purpose we created 18 different types of dynamic smiles by varying, based on the Facial Action Coding System (FACS, [33]), the degree of activation in three regions of avatar faces: the crow’s feet around the eyes (AU6), the pulling up of the corners of the lips (AU12), and the opening of the mouth and lips (AU25, AU26). One additional smile also included light frowning (AU1, AU4). We hypothesized that each of these facial actions contributes to the perception of smile authenticity, and more specifically that participants would rely less on the Duchenne marker to judge dynamic smile authenticity, than did participants in earlier studies that used static smile stimuli. Although higher authenticity ratings were expected for the strongest smiles, we focused on participants’ subjective ratings of authenticity, instead of defining smile authenticity a priori.

### The Role of Facial Mimicry

Studying how people judge that a smile is true versus false may constitute an ideal case to examine the general mechanisms underlying the recognition of facial expressions.

According to theories of embodied cognition, *our own* emotional information is processed through somatovisceral and motoric re-experiencing [34]–[36]. This theoretical proposition has a long history in philosophy and scientific psychology [5], [37]. The more recent “facial feedback hypothesis” emphasizes the role of facial movements in shaping emotions, and specified a correspondence between facial expressions and subjective experience [38]–[40].

Embodiment may also play a role in understanding the emotions, intentions, and behaviors of *other* people. After being described in the 18^th^ century ([41], p.5), motor mimicry of another person’s expression of affect was suggested by Theodor Lipps to support empathy [42]. Mimicry of facial expressions, but also gestures, postures, and other body movements, has since been shown to occur, across several studies, in adults (e.g., [43]–[45]), and in newborn infants [46], [47]. Automatic facial mimicry is difficult to suppress voluntarily [48], and may occur even in the absence of conscious perception of the stimulus face [49].

Summarizing the view of most embodiment theories, Hatfield et al. [50] proposed in their two-stage model that emotions are based on the facial feedback resulting from facial mimicry. This claim is now supported by a number of empirical findings [35], [38]. For example, blocking facial mimicry slows the recognition of positive and negative facial expressions [51], impairs the distinction between true and false smiles [4], [52], delays the perception of the offset of happy and sad facial expressions [53], and interferes with the recognition of happiness [54]. Furthermore, interfering with the functioning of the Corrugator muscle (involved in frowning) with injections of botulinum toxin decreases amygdala responses to angry faces, and reduces the functional coupling between the amygdala and brain stem regions implicated in autonomic emotional responses [55].

These and other findings suggest that individuals rely on feedback from facial mimicry to process facial expressions, and that emotion recognition becomes slower and less accurate when facial mimicry is blocked. However, other studies of this link have failed to find support for the hypothesized mediation of facial mimicry in the facial expression recognition process [56].

A sensitive test of the hypothesis that facial mimicry supports the decoding of facial expressions may be obtained by asking participants to judge the authenticity of various similar types of smiles. Interestingly, only one prior study has so far partially addressed this question. In it, participants were exposed to experimental blocks of Duchenne and non-Duchenne smiles interspersed with neutral faces. As indicated by measures of facial EMG, participants reacted with greater contraction of their periocular and cheek muscles to Duchenne smiles than to neutral faces. However, reactions to non-Duchenne smiles were not distinguishable from reactions to neutral faces. The fact that participants mimicked true but not false smiles may be due to the use of static pictures as stimuli, which have been shown to be less powerful than dynamic stimuli in generating facial mimicry [26]. Unfortunately, the study did not test if the accuracy of participants’ decoding of the smiles was related to the degree to which they mimicked them.

A secondary aim of the present research was to test the hypothesis, derived from embodied emotion theories, that facial mimicry contributes to the recognition and interpretation of facial expressions of emotion. Facial mimicry was measured with facial EMG, a technique used to assess changes in muscular contractions, even ones that are invisible to the eye [57], from the areas overlying four facial muscles: 1) the Corrugator Supercilii muscle, which when contracted results in frowning, 2) the Orbicularis Oculi muscle, which creates crow’s feet wrinkles around the eyes, and thus forms the so-called Duchenne marker, 3) the Zygomaticus Major muscle, which is the main smile muscle that pulls the corners of the mouth up and backwards, and 4) the Masseter muscle, which when relaxed contributes to the dropping of the jaw.

We hypothesized that participants would mimic the dynamic smiles – as revealed by greater activation of the participants’ facial muscles to trials containing higher levels of the corresponding AUs in the stimuli – and that their mimicry would mediate the effect of smile intensity in the stimulus on perceived authenticity. We particularly expected activity in the Zygomaticus Major and Orbicularis Oculi muscles to be positively correlated, and the Corrugator muscle to be negatively correlated with ratings of authenticity. This prediction was based on the typical anti-correlation between Corrugator vs. Zygomaticus and Orbicularis Oculi muscles during smiling [58]. In addition, increased Zygomaticus contraction has been reported in response to pictures of positive valence [59], while Corrugator contraction is increased for negative and decreased for positive visual stimuli [59]–[61].

## Methods

### Participants

Thirty-one participants (11 men, mean age = 22.1, SD = 3) were recruited at the University of Geneva, and participated in exchange of 20 Swiss francs (about $20). All were right-handed, with normal or corrected to normal vision, and free of neurological or psychiatric disorders. All participants gave written informed consent. The study was approved by the ethical committee of the faculty of psychology at the University of Geneva.

### Stimuli

**Figure 1:**
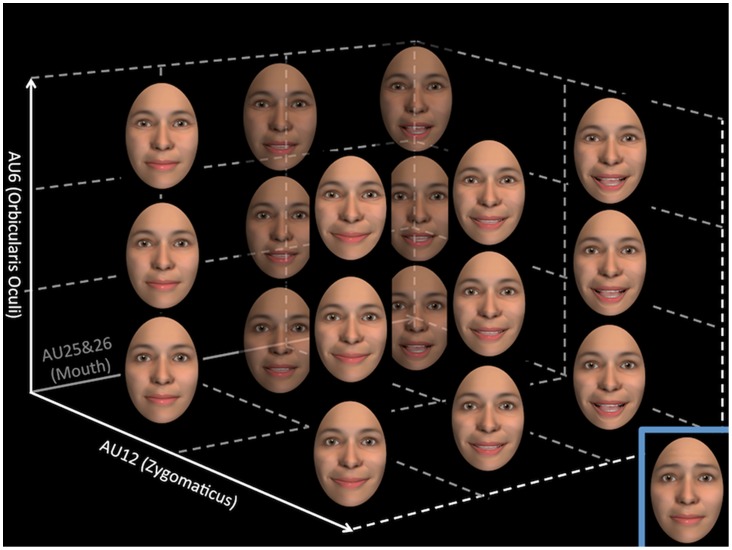
Representation of the apex of the 18 different types of smiles, which were created by varying the activation intensity of 3 muscle region factors: Zygomaticus (AU12), Orbicularis Oculi (AU6), Mouth and lips opening (AU25&26). An additional 19^th^ Mixed smile (lower right of the figure) was created by adding activation of the Corrugator (AU1&4) to weak smiles.

Stimuli were created and verified by a certified and experienced FACS coder (S.W.) and consisted of 8 avatar faces (four males) created with FaceGen (www.facegen.com). The faces were animated using FACSGen [62], a software based on the Facial Action Coding System [33] and used to create still pictures as well as videos of faces with dynamically changing facial expressions. Importantly, in FACSGen each Action Unit can be manipulated individually. Short video clips with 19 different types of smiling expressions were created for each avatar face by varying the activation intensity of four smile components composed of six of FACS’ action units (AUs). The AUs used and their respective levels of activation (in terms of FACSGen’s scales) are shown in Table 1, example photos of the 19 smiles at their apex are shown in Figure 1.

**Table 1:** Activation of involved Action Units (AUs), Smile-Stimulus scores and average authenticity rating, for the 19 stimulus types.

		Smile	Eyes	Mouth opening		Frown			
N°	Stimulus name	AU12	AU6	AU25	AU26	AU1	AU4	Smiling score	Authenticity (SD)
1	Weakest	50%	0%	0%	0%	0%	0%	50	−0.41 (0.72)
2	Mixed	50%	0%	0%	0%	50%	50%	0	−0.79 (0.63)
3		50%	50%	0%	0%	0%	0%	100	−0.41 (0.52)
4		50%	50%	50%	50%	0%	0%	100	−0.25 (0.39)
5		50%	50%	100%	100%	0%	0%	100	−0.24 (0.38)
6	Weakest with AU6	50%	100%	0%	0%	0%	0%	150	−0.49 (0.61)
7		50%	100%	50%	50%	0%	0%	150	−0.40 (0.52)
8		50%	100%	100%	100%	0%	0%	150	0.16 (0.49)
9		50%	0%	50%	50%	0%	0%	50	−0.43 (0.48)
10		50%	0%	100%	100%	0%	0%	50	−0.21 (0.65)
11		100%	0%	0%	0%	0%	0%	100	0.24 (0.65)
12		100%	50%	0%	0%	0%	0%	150	0.48 (0.58)
13		100%	50%	50%	50%	0%	0%	150	0.31 (0.36)
14		100%	50%	100%	100%	0%	0%	150	0.67 (0.52)
15		100%	100%	0%	0%	0%	0%	200	0.17 (0.72)
16		100%	100%	50%	50%	0%	0%	200	0.33 (0.51)
17	Strongest	100%	100%	100%	100%	0%	0%	200	0.81 (0.59)
18		100%	0%	50%	50%	0%	0%	100	0.11 (0.58)
19	Strongest No AU6	100%	0%	100%	100%	0%	0%	100	0.36 (0.68)

All videos were 2 seconds long and had a frame rate of 25 frames per second. Each one started with a neutral expression, which changed linearly into a smiling expression until it reached its peak after 1 second. The apex expression then remained on the screen for another second.

The videos were exported from FACSGen as still pictures onto which a black frame with a central oval-shaped hole was superimposed, using Photoshop (Adobe Systems Inc.). This was done to show only the face while hiding the hair and face contours (for a similar procedure see [63], [64]). The pictures with the superimposed black frame were then retransformed into 2-second-long video clips.

All videos thus displayed a neutral face rapidly turning into a weak or strong smile (AU12 was always present) with either open or closed lips and mouth (AU25 and AU26), with or without crow’s feet (AU6), and either without, or - in a small number of cases – with Corrugator activation leading to frowning (AU1 and AU4). Example videos from one male avatar are available at http://cms2.unige.ch/cisa/stimulus_videos/example_videos_of_19_smiles.zip.

### Procedure

After having provided informed consent, participants had EMG electrodes attached and were seated in a comfortable chair in a dimly lit room, at a distance of approximately 70 cm from a computer screen. They then completed two tasks, the order of which was held constant across participants.

First, a short task required rating the facial attractiveness of the eight avatar identities, presented as still pictures with a neutral expression. Specifically, over eight trials a fixation cross (2 sec) was followed by the picture of an avatar’s face (2 sec), and then by a scale on which participants rated the attractiveness of the face from 1 (*not at all attractive*) to 100 (*very attractive*). On each trial, the scale first appeared with a response set at the midpoint (50), and participants submitted their ratings by moving the mouse either to the right or left, and finally clicking the left mouse button in order to advance to the next trial. Stimulus order was random and differed for all participants.

The main task, which always followed the attractiveness ratings, required participants to watch and rate the authenticity of 152 different 2-second-long smile videos. True smiles were defined as “the type of smile a person makes spontaneously when she is happy, joyful, or amused”. The definition of a false smile was “the type of smile a person makes voluntarily when she wants to be polite, but does not actually feel very happy, joyful, or amused”. Examples were also provided orally by one of the experimenters, who noted that “a true smile is likely to occur when two good old friends, who have not seen each other for a long time, meet again” and “a false smile is likely to occur when a person receives a gift she actually dislikes, but still wants to be polite.”

Trials were composed of a fixation cross (average duration = 2.5 sec, range = 2–3 sec), a video (2 sec), and a rating scale. The rating scale was anchored by 1 (*not at all authentic*) and 100 (*very authentic*). The order of stimuli was different for each participant and semi random, with a maximum of three successive stimuli with the same level of activation of AU6 (Orbicularis Oculi). The task was divided into two blocks of 76 stimuli each, and participants were free to rest briefly between blocks.

At the end of the experiment, electrodes were removed and participants were debriefed.

### Electrophysiological Recording and Data Reduction

Using a BIOSEMI (www.biosemi.com) ActiveTwo amplifier system with Ag/AgCl active electrodes and a sampling rate of 1024 Hz, facial EMG was recorded according to guidelines [65] over the left Corrugator Supercilii (CS), Orbicularis Oculi (OC), Zygomaticus Major (ZM), and Masseter (MA) muscles. Off-line, using MATLAB (www. mathworks.com) and the EEGLAB toolbox [66], EMG data were put into bipolar montage, band-pass filtered between 20 and 200 Hz, full-wave rectified, segmented from 1 second before to 2 seconds after stimulus onset (SO), and smoothed with a 40 Hz low-pass filter (smoothing is commonly applied to rectified EMG data, e.g. see [67]).

For each participant, we excluded trials on which the average amplitude in the baseline period (−1 sec to SO) of any of the four muscles exceeded by more than 2 SDs the average amplitude over all trials’ baselines of the respective muscle (excluded trials over all participants *M* = 20.6, *SD* = 5.5 – approximately 13% of the total number of trials). Then, in order to compare across muscles, data from SO to 2 seconds later were expressed as percentage of the average of the 1-second long baseline (for a similar procedure see [68], [69]).

### Data Analysis

**Figure 2:**
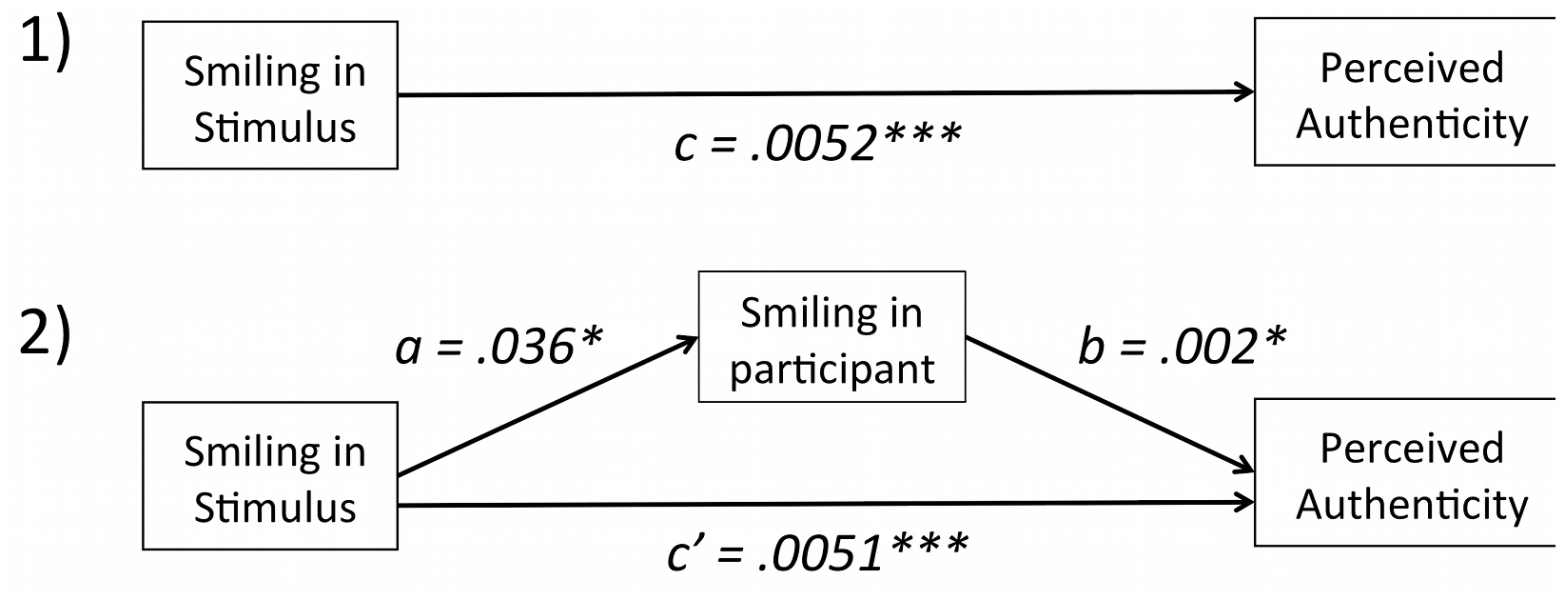
Diagrams of 1) expected effects of smiling intensity in the stimulus on perceived smile authenticity, and 2) mediation of this effect by participants’ facial mimicry. Smiling in the stimulus significantly predicted both smiling in the participant (a) and perceived authenticity (c). Moreover, authenticity was predicted by smiling in the participant (b). However, no clear signs of mediation (path c’<path c) were found.

Ratings of facial attractiveness (first task) and of smile authenticity (main task) were transformed to z-scores (i.e. each rating, minus that participant’s mean, divided by that participant’s SD), in order to account for possible differences in the use of the rating scales across participants. To test the hypothesis that stronger smiles are perceived as more authentic (see Figure 2, path c), we computed for each trial a *Smile-Stimulus score* by adding the levels of activation of AU6 and AU12, and subtracting the activation of AU1&4. The logic behind this equation was that smiles typically elicit increased activity in AU12 and AU6, along with decreased activity in AU1&4 [58]. A linear mixed model with random intercepts and slopes for both participants and avatars was then fit to predict authenticity ratings from smiling scores. F-tests were computed, and degrees of freedom were estimated using the Satterthwaite approximation. A similar model was fit to predict authenticity from attractiveness ratings.

To examine more specifically how AUs affect the perception of smile authenticity, ratings of authenticity were analyzed in a repeated-measures analysis of variance (rmANOVA) with the factors AU12 (2 levels: 50 and 100%), AU25&26 (3 levels: 0, 50, and 100%), and AU6 (3 levels: 0, 50, and 100%). Authenticity ratings of stimuli including AU1&4 were analyzed in a separate rmANOVA with the factor Frown (2 levels: 0, and 50%) – the corresponding number of trials without frowning was randomly selected.

EMG data were analyzed in various ways according to hypotheses:

First, a *Smile-Participant* score was calculated by adding the EMG activity in the 2^nd^ time-window of the Zygomaticus and Orbicularis Oculi muscles, and by subtracting that of the Corrugator muscle. After removing 140 trials (3.5 %) in which the EMG exceeded the mean by more than two standard deviations, a linear mixed model with random intercepts and slopes for both participants and avatars was fit to predict the Smile-Participant scores from the Smile-Stimulus scores, to test the hypothesis of facial mimicry (Figure 2, path a). The same model was fit using attractiveness ratings as the independent variable.

Second, we conducted for each muscle an rmANOVA with the factors AU12 (2 levels: 50 and 100%), AU25&26 (3 levels: 0, 50, and 100%), AU6 (3 levels: 0, 50, and 100%), and Time (2 levels: 0−1 s, and 1−2 s).

Third, in order to test whether the perception of smiles with specific configurations of AUs results in facial mimicry, we averaged the trials for 5 types of smiles with the most extreme characteristics (see Table 1 for the corresponding AUs). The rationale behind this selection was to include the highest and the lowest level of activation of the AUs corresponding to the sampled EMG muscles. An rmANOVA was computed with the factors Stimulus (5 levels: Strongest, Strongest No AU6, Weakest, Weakest With AU6, Mixed), Muscle (4 levels: CS, OC, ZM, MA) and Time (2 levels: 0−1 s, and 1−2 s). Planned contrasts as well as exploratory post-hoc tests accompany this ANOVA.

Finally, we tested the hypothesis that facial mimicry mediates the effect of smiling in the stimulus on authenticity ratings. Following the procedure by Baron and Kenny [70], we compared the estimates for the model predicting authenticity from smiling in the stimulus by itself (Figure 2, path c), vs. controlling for smiling in the participant (Figure 2, path c’).

Statistical analyses were conducted in SPSS and R. Standardized effect sizes are provided as partial Eta squared (η_p_^2^). Sphericity violations in ANOVAs underwent Greenhouse–Geisser correction, in which case corrected *p* values but uncorrected degrees of freedom are reported. Data are available on request.

## Results

### Ratings

**Figure 3:**
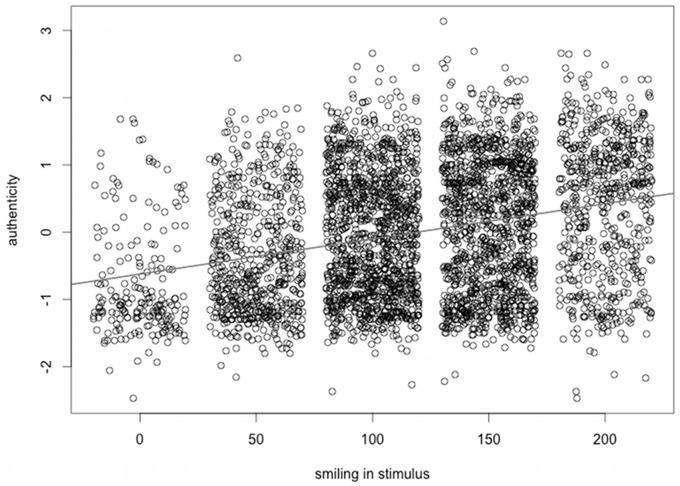
Intensity of smiling in the stimulus significantly predicted judgments of authenticity. Data on the x-axis was jittered to improve display.

A linear mixed effects model fitted to predict authenticity ratings from Smile-Stimulus scores (Figure 2, path c, and Figure 3) was significant (*estimate* = .0052; *F*(1, 7.9) = 25.74, *p*<.001), suggesting, as expected, that perceived authenticity increased for stronger smiles. In contrast, attractiveness ratings did not predict authenticity judgments (*F*(1, 5.9) = 1.96, *p* = .21).

**Figure 4:**
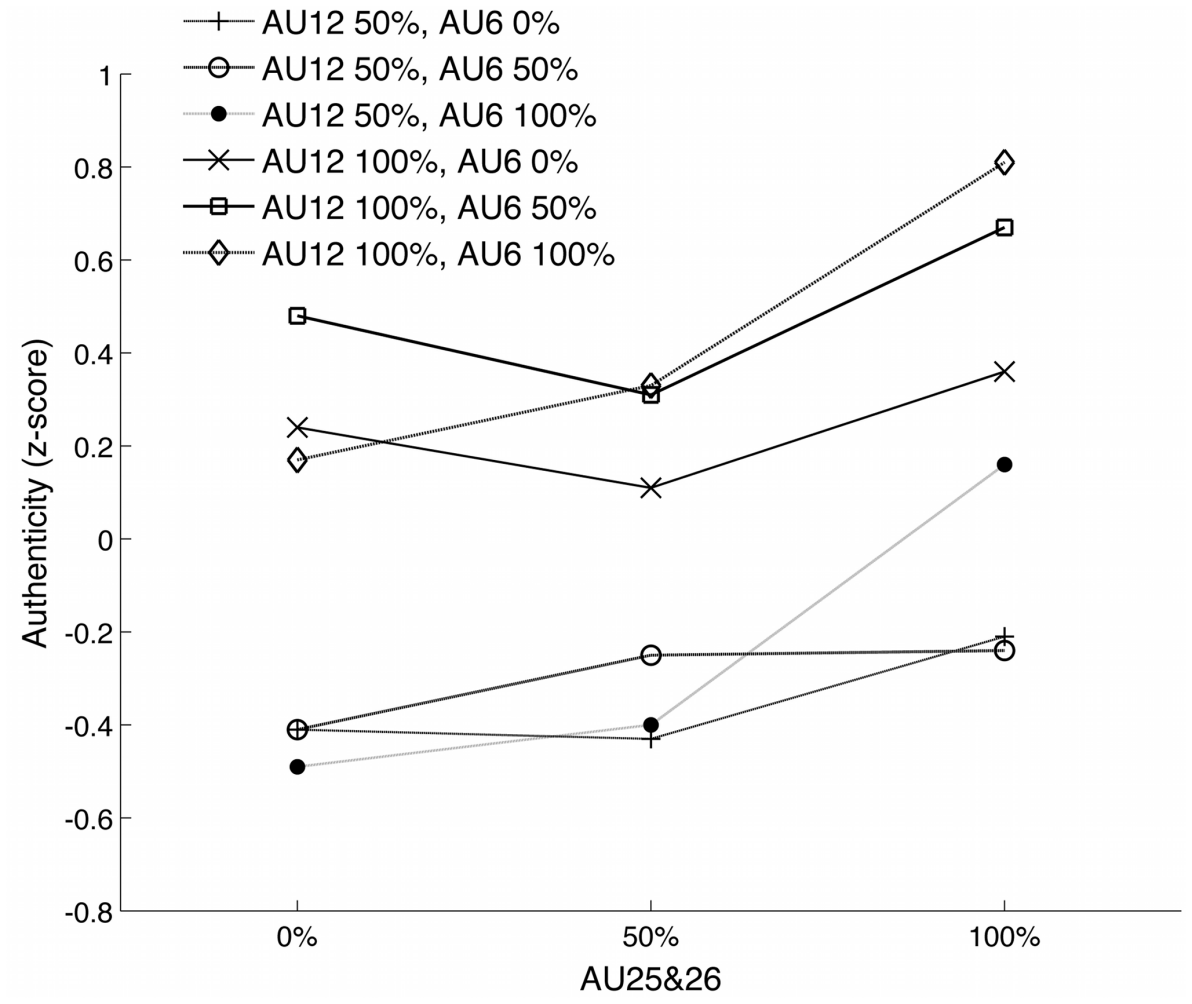
Ratings of authenticity, which resulted in a triple interaction. The leading factor in making a smile appear authentic was the intensity of AU12, as evident in the two vertically stacked clusters of lines on the graph. Importantly, although the degree of AU6 activation increased perceived authenticity, it did so in interaction with AU12 and AU25&26.

The contribution to this effect of each single AU was further analyzed in an ANOVA carried out on the ratings of authenticity with the factors AU12, AU25&26, and AU6 (see Table 2). Exclusion of male participants yielded the same results, with exception of the AU12 X AU25&26 interaction, which no longer reached significance (*F*(2,38) = 2.54, *p*<.11, *η_p_^2^* = .12). Post-hoc tests indicated that there were higher ratings of authenticity for 100% (*M* = −.319, *SD* = .18) than 50% (*M* = .386, *SD* = .22) of AU12 (*p*<.001), and for 100% (*M* = .255, *SD* = .31) compared to 50% (*M* = −.048, *SD* = .16) or 0% (*M* = −.106, *SD* = .35) of AU25&26 (both *p*<.005). Moreover (see Figure 4), in the case of weak smiles (AU12 = 50%), ratings of authenticity increased linearly from 0% (*M* = −.512, *SD* = .37) over 50% (*M* = −.351, *SD* = .25) to 100% (*M* = −.094, *SD* = .30) of AU25&26 (all *t*>2.16, all *p*<.04). However, ratings did not differ between 0% (*M* = .30, *SD* = .49) and 50% (*M* = −.25, *SD* = .26) of AU25&26 in the case of 100% AU12 (*p* = ns), which were smaller than ratings for 100% (*M* = .60, *SD* = .44) of AU25&26 (both *p*<.04). The AU25&26 X AU6 interaction showed that while 100% of AU25&26 always leads to greater perceived smile authenticity compared to 0% of AU25&26, these differences in authenticity ratings become even bigger in combination with a strongly contracted Orbicularis Oculi (AU6 = 100% Eyes).

**Table 2:** Results of the rmANOVA on Authenticity ratings with factors AU12, AU25&26, and AU6.

Effect	SS (Error)	*Df* (Error)	F	η_p_^2^	p
AU12	69.20 (21.44)	1(30)	96.9	.764	< .001***
AU25&26	13.96 (42.99)	2(60)	9.74	.245	.002**
AU6	3.82 (60.73)	2(60)	1.88	.059	.160
AU12 X AU25&26	1.02 (6.63)	2(60)	4.61	.133	.014*
AU12 X AU6	.36 (4.45)	2(60)	2.39	.074	.1
AU25&26 X AU6	3.89 (10.81)	4(120)	10.79	.265	<.001***
AU12 X AU25&26 X AU6	.93 (7.47)	4(120)	3.73	.111	.010*

*Note*.***p<.001;

**p<.01;

*p<.05.

As expected, the ANOVA comparing trials with and without frowning resulted in a significant main effect of the factor Frown (*F*(1,30) = 39.63, *p*<.001), due to higher ratings of authenticity for smiles with 0% frown (*M* = .04, *SD* = .36), than with 50% frown (*M* = −.77, *SD* = .62).

An online study was carried out in a separate sample of 21 participants from the University of Wisconsin, Madison, to test for differences in perceived realism of the avatar smiles, and more specifically whether failure to obtain a main effect of AU6 in the authenticity judgments may have been caused by a lack of realism of the smiles with Duchenne marker. Participants rated all stimulus videos for their degree of realism – defined as “how likely is it that you would see a real person showing that expression?” – using a 100-point Likert scale. Realism scores were z-scored, averaged across avatar faces and participants. Realism and authenticity scores were significantly correlated (*r* = .83, *p*<.001), indicating that the smiles with the highest authenticity scores were also being perceived as more realistic. Realism scores were also analyzed in an rmANOVA, resulting in a main effect of AU12 (*F*(1,20) = 23.12, *p*<.001), a trend-like effect of AU6 (*F*(2,40) = 3.73, *p* = .056); a significant AU12 X AU6 interaction (*F*(2,40) = 3.51, *p* = .046); and a significant AU12 X AU25&26 X AU6 interaction (*F*(4,80) = 2.76, *p* = .043). Differences between levels of AU6 were tested with paired-samples t-tests, showing that smiles with 50% and 100% of AU6 had similar levels of realism (*t*(20) = 1.4, *p* = .18), while smiles with 0% AU6 were perceived as significantly less realistic compared to those with 50% AU6 (*t*(20) = 3.8, *p* = .001). The results of this realism rating study suggest that the lack of a main effect of AU6 in authenticity judgments (see above) is unlikely to be due to a lack of realism in smiles with Duchenne marker.

### EMG

To test if participants mimicked the perceived smiles, we first fitted a linear mixed effects model to predict Smile-Participant scores from Smile-Stimulus scores (Figure 2, path a). This model was statistically significant (*estimate* = .036; *F*(1, 28.31) = 5.32, *p* = .028), confirming that mimicry of the perceived smiles did occur. In contrast, attractiveness ratings did not predict smiling in the participant (*F*(1, 28.63) = .94, *p* = .341).

To investigate the mimicry effect in greater detail, we computed for each muscle an rmANOVA over all stimuli with the factors AU12 (50 and 100%), AU25&26 (0, 50, and 100%), AU6 (0, 50, and 100%), and Time (0−1 s, and 1−2 s).

For the Corrugator muscle the analysis produced a main effect of AU12 (*F*(1,30) = 9.42, *p* = .005, *η_p_^2^* = .24) due to smaller activation with increasing AU12; a trend-like effect of AU25&26 (*F*(2,60) = 2.55, *p* = .086, *η_p_^2^* = .08), due to linearly decreasing Corrugator activation with increasing opening of the stimuli’s mouth and lips; and significant AU6 X Time (*F*(2,60) = 4.41, *p* = .016, *η_p_^2^* = .13) and AU12 X Time interactions (*F*(1,30) = 10.57, *p* = .003, *η_p_^2^* = .26). No other effects were significant (all *F*<1.89, all *p*>.1, ns).

For the Orbicularis Oculi muscle we found a significant effect of Time (*F*(1,30) = 24.378, *p*<.001, *η_p_^2^* = .45) due to higher EMG activation in the second time window; a trend-like main effect of AU25&26 (*F*(2,60) = 2.65, *p* = .079, *η_p_^2^* = .08), and two marginally significant interactions for AU12 X AU6 X AU25&26 (F(4,120) = 2.03, *p* = .095, *η_p_^2^* = .06), and AU12 X Time (*F*(1,30) = 3.39, *p* = .075, *η_p_^2^* = .1). No other effects were significant (all *F*<2.34, all *p*>.1, ns).

The same rmANOVA for the Zygomaticus muscle revealed a marginally significant effect of AU6 (*F*(2,60) = 3.09, *p* = .062, *η_p_^2^* = .09); a significant effect of AU12 (*F*(1,30) = 4.3, *p* = .047, *η_p_^2^* = .12) due to greater Zygomaticus contraction to 100% AU12; a significant effect of Time (*F*(1,30) = 19.92, *p*<.001, *η_p_^2^* = .4) due to higher EMG in the second time window; and a trend-like AU12 X Time interaction (*F*(1,30) = 3.38, *p* = .076, *η_p_^2^* = .1). No other effects were significant (all *F*<2.25, all *p*>.1, ns).

For the Masseter muscle we found a significant effect of AU6 (*F*(2,60) = 4.02, *p* = .023, *η_p_^2^* = .12), due to greater Masseter contraction for stimuli with 50% than 0% of AU6 (*p*<.05); and a significant effect of Time (*F*(1,30) = 6.8, *p* = .014, *η_p_^2^* = .18), due to greater Masseter activation in the second time window (*p*<.05). No other effects were significant (all *F*<1.99, all *p*>.1, ns).

Results were similar when male participants were excluded from the analyses, with a few exceptions: For the Orbicularis Oculi a main effect of AU12 just fell short of significance (*F*(1,19) = 4.27, *p* = .053, *η_p_^2^* = .18), with higher EMG to stimuli containing more of AU12. For the Zygomaticus, an even stronger effect of AU12 was found (*F*(1,19) = 10.52, *p* = .004, *η_p_^2^* = .36), but the effect of AU6 no longer reached significance (*F*(2,38) = 2.37, *p* = .11, *η_p_^2^* = .11).

**Figure 5:**
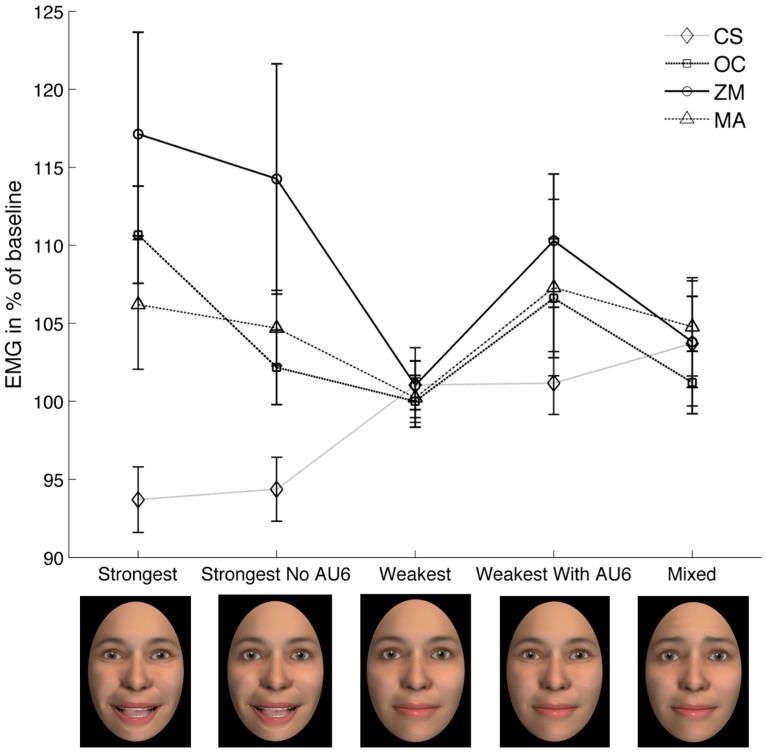
Average (and standard error) EMG for the four sampled muscles, across five stimulus sub-types, averaged over the entire epoch after SO. A Stimulus X Muscle X Time ANOVA resulted in a significant Stimulus X Muscle interaction (see main text for details, and Table 3 for post-hoc tests). CS = Corrugator Supercilii, OC = Orbicularis Oculi, ZM = Zygomaticus Major, MA = Masseter.

In order to further test the hypothesized presence of facial mimicry, by directly comparing EMG amplitude of each muscle in response to the highest and lowest levels of the corresponding AUs, we selected a subset of five types of smiles (see Figure 5 for results, see Table 1 for descriptions of the AUs involved), and analyzed it in an rmANOVA with the factors Stimulus (Strongest, Strongest No AU6, Weakest, Weakest With AU6, Mixed), Muscle (CS, OC, ZM, MA), and Time (1^st^, 2^nd^ second). This produced significant main effects of Muscle (*F*(3,90) = 4.73, *p* = .018, *η_p_^2^* = .14), and Time (*F*(1,30) = 8.31, *p* = .007, *η_p_^2^* = .22), as well as significant Stimulus X Muscle (*F*(12,360) = 3.35, *p* = .007, *η_p_^2^* = .1) and Muscle X Time (*F*(3,90) = 3.92, *p* = .024, *η_p_^2^* = .12) interactions. No other effects were significant (all *F*<2, all *p*>.1). The same main and interaction effects were found when excluding male participants, with exception of the main effect of Muscle (*F*(3,57) = 2.18, *p* = .12, *η_p_^2^* = .10).

The crucial Stimulus by Muscle interaction was further explored using post-hoc t-tests (uncorrected, see Table 3), which overall confirmed the facial mimicry hypothesis, by showing that 1) Corrugator contraction was highest to Mixed smiles – the only type of smiles to contain AU1&4; 2) Orbicularis Oculi contraction was highest when AU6 was strongly activated in the stimuli; 3) Zygomaticus activation was greater for smiles including the highest level of AU12; 4) the Masseter was more active to trials containing an open mouth.

**Table 3:** Planned contrasts (P) and post hoc comparisons involving the 5 selected types of smiles.

Muscle	Comparison	Mean1(SD)	Mean2(SD)	t-value	p-value
Corrugator	(P) Mixed vs. all other smiles	103.7 (21.4)	97.6 (9.2)	1.9	.067
	Mixed vs. Strongest	103.7 (21.4)	93.7 (10.4)	2.95	.006**
	Mixed vs. Strongest_No_AU6	103.7 (21.4)	94.4 (10.7)	2.53	.017*
	Mixed vs. Weakest	103.7 (21.4)	101 (12.9)	.72	.477
	Mixed vs. Weakest_With_AU6	103.7 (21.4)	101.2 (10.8)	.87	.388
Orbic. Oculi	(P) Strongest & Weakest_With_AU6 vs. Weakest & Strongest_No_AU6 & Mixed	108.6 (12.6)	101.1 (8.3)	3.36	.002**
	Strongest vs. Strongest_No_AU6	110.7 (15.4)	102.2 (12.4)	2.60	.014*
	Strongest vs. Weakest	110.7 (15.4)	100 (8)	3.63	.001**
	Strongest vs. Mixed	110.7 (15.4)	101.2 (9.7)	3.30	.002*
	Weakest_With_AU6 vs. Strongest_No_AU6	106.6 (19.3)	102.2 (12.4)	1.24	.223
	Weakest_With_AU6 vs. Weakest	106.6 (19.3)	100 (8)	1.93	.063
	Weakest_With_AU6 vs. Mixed	106.6 (19.3)	101.2 (9.7)	1.48	.150
Zygomaticus	(P) Strongest & Strongest_No_AU6 vs. Weakest & Weakest_With_AU6 & Mixed	115.7 (25.2)	105.1 (12.3)	2.56	.016*
	Strongest vs. Weakest	117.1 (34.1)	101 (7.6)	2.67	.012*
	Strongest vs. Weakest_With_AU6	117.1 (34.1)	110.3 (22.9)	1.07	.294
	Strongest vs. Mixed	117.1 (34.1)	103.8 (13.7)	2.29	.029*
	Strongest_No_AU6 vs. Weakest	114.3 (37)	101 (7.6)	2.05	.049*
	Strongest_No_AU6 vs. Weakest_With_AU6	114.3 (37)	110.3 (22.9)	.52	.608
	Strongest_No_AU6 vs. Mixed	114.3 (37)	103.8 (13.7)	1.78	.087
Masseter	(P) Strongest & Strongest_No_AU6 vs. Weakest & Weakest_With_AU6 & Mixed	105.5 (12.5)	104.1 (12.7)	.55	.583
	Strongest vs. Weakest	106.2 (20.5)	100.2 (5.2)	1.71	.098
	Strongest vs. Weakest_With_AU6	106.2 (20.5)	107.3 (30.8)	-.2	.844
	Strongest vs. Mixed	106.2 (20.5)	104.8 (13.9)	.41	.682
	Strongest_No_AU6 vs. Weakest	104.7 (10.7)	100.2 (5.2)	2.15	.040*
	Strongest_No_AU6 vs. Weakest_No_AU6	104.7 (10.7)	107.3 (30.8)	-.43	.671
	Strongest_No_AU6 vs. Mixed	104.7 (10.7)	104.8 (13.9)	-.03	.975

*Notes*:*p<.05.

**p<.01.

***p<.001.

Finally, planned contrasts (see Table 3) compared the EMG of each muscle in response to trials with and without the presence of the corresponding AU(s). The expected specificity of each muscle’s response to stimuli containing the corresponding muscle contraction was found for the Orbicularis Oculi (*t* = 3.36, *p* = .002) and Zygomaticus muscles (*t* = 2.56, *p* = .016), and reached trend level for the Corrugator (*t* = 1.9, *p* = .067). However, the degree of Masseter activation did not correspond to the amount of AU25&26 contained in the stimuli (*t* = .55, *p* = .583).

To address the hypothesis that participants’ facial mimicry during smile perception mediates judgments of smile authenticity, we fitted a linear mixed effects model to predict authenticity ratings from Smile-Stimulus scores, controlling for the effect of Smile-Participant scores on authenticity (estimates of the slopes for the random effects of subject and avatar were dropped in this model in order to allow for convergence). If mediation exists, path c’ in Figure 2 should be smaller than path c [70]. The coefficient of path c’ (*estimate* = .0052, *SE* = .002; *F*(1, 3904.71) = 363.25, *p*<.001) remained unchanged compared to the model not controlling for the mediator (Figure 2 path c, *estimate* = .0052, *SE* = .001). Therefore, the results do not confirm the mediation by mimicry hypothesis.

## Discussion

The present experiment attempted to isolate the features of smiles that are diagnostic for judgments of authenticity, and to test a hypothesized role of facial mimicry in decoding smiles. The main results were: 1) overall stronger smiles were judged as being more authentic, but contraction of the Duchenne marker only predicted judgments of authenticity in combination with the other components of the smile, 2) participants showed clear signs of facial mimicry of the avatar smile stimuli, 3) mimicry predicted authenticity judgments, but 4) significant mediation was not observed. In the following, we discuss each of these findings in turn.

Our primary goal was to uncover the features or feature combinations that determine judgments of smile authenticity. In light of recent studies that have demonstrated that the Duchenne marker is less diagnostic of perceived authenticity in dynamic compared to static smiles [71], and the fact that decoding dynamic versus static smiles may rely on partially separate neural circuits [29], [30], we constructed and employed a set of highly controlled dynamic smiles expressed by avatars (Figure 1, and Table 1). Importantly, our stimulus set also included intermediate levels of the Duchenne marker, while most previous studies only focused on the presence/absence of this smile feature.

Findings showed that stronger smiles were perceived as more authentic and – in a separate rating study – as more realistic. In addition, perceived authenticity was influenced by all three face areas that were varied in the stimuli (see Figure 4). Importantly, AU6, the Duchenne marker, did not convey authenticity by itself, but only in interaction with AU12, the smile muscle, and the opening of the mouth and lips. This finding suggests that when dynamic instead of static smiles are presented, and the different AUs of smiles are controlled, people do not solely rely on the presence or absence of the Duchenne marker to infer smile authenticity. Instead, they make use of all the smile features, including the degree of lip and mouth opening. Importantly, smiles with 50% of AU6 were perceived as realistic as those with 100% AU6, and as significantly more realistic than those without AU6. This excludes the possibility that failure to find a significant effect of AU6 in authenticity judgments may have been caused by the unrealistic appearance of smiles with higher levels of AU6.

Our secondary goal was to explore the hypothesis that facial mimicry contributes to the process of decoding another person’s facial expression [34], [35], [42], [50], especially when expressions are similar to each other, so that they cannot be readily decoded from stereotypical features [6], and when the processing goal is more complex than a simple act of categorization of prototypic expressions. In what is the first test of this kind, we used a stimulus set of highly similar smiles, recorded the EMG from facial areas targeting four muscles, and were able to show that smiling intensity of the stimulus predicts both facial mimicry and perceived authenticity.

Facial mimicry was confirmed by a significant linear fit of a linear mixed effects model predicting Smile-Participant from Smile-Stimuli scores (Figure 2, path a). Second, separate analyses for each muscle revealed a significant main effect of AU12 for the Zygomaticus and Corrugator muscle, indicating facial mimicry in terms of increased Zygomaticus contraction and decreased Corrugator contraction (these muscles are anti-correlated during smiling, e.g see [58]). Also, analyses carried out on a sub-set of the five most characteristic stimuli revealed a significant Stimulus by Muscle interaction (see Figure 5). Here, exploratory post hoc tests (see Table 3) showed that 1) the Corrugator muscle contracted most in response to “Mixed” smiles containing AUs 1 and 4; 2) the Orbicularis Oculi contracted more in response to smiles with the highest level of AU6, compared to those with the lowest; 3) the Zygomaticus contracted more in response to smiles containing 100% compared to 50% of AU12 activation – with the exception of “Weakest With AU6” smiles. Similarly, planned contrasts confirmed that, as expected, the Orbicularis Oculi and Zygomaticus muscles were significantly more activated on trials that included the highest compared to trials containing the lowest degree of their corresponding AUs (i.e. AU6 and AU12, respectively), and the Corrugator showed a strong trend for the same type of response specificity, with greater frowning in response to stimuli containing frowning. Only the Masseter muscle did not show the expected (nor any other significant) facial mimicry pattern, with greater contraction to smiles with closed compared to open jaw, possibly due to cross talk from the Zygomaticus muscle [72], [73]. Thus, the hypothesis that participants show facial mimicry during smile perception was confirmed by the findings that the Corrugator, Orbicularis Oculi, and Zygomaticus muscles overall showed the expected pattern of increased EMG activity to smiles containing activation of the corresponding AUs.

Embodiment theories propose that facial mimicry is a low-level motor process that can generate or modify emotional processes via facial feedback [35], [55]. However, other scholars favor the view that facial expressions are the downstream reflection of an internally generated emotion [74], [75], and therefore play at best a minor role at a later stage of the emotion generation process [76]. The main critique of the embodiment view is based on the observation that, in addition to their well-documented role in facial mimicry, the Zygomaticus and Corrugator muscles respond, respectively, to positive and negative emotional stimuli not containing facial expressions [59]. However, the Orbicularis Oculi muscle is not clearly associated with positive or negative emotions and contracts, for example, during smiling (producing crow’s feet) as well as during a startle reflex in response to a sudden loud noise [73]. Most studies on facial mimicry record the Zygomaticus and Corrugator muscles, but do not monitor the Orbicularis Oculi muscle. Moreover, they seldom control for the degree of Duchenne marker activation in the stimulus faces, independently of the other smile features.

Therefore, mimicry of the Orbicularis Oculi muscle observed in the present experiment provides a relatively rare opportunity to shed light on the question whether facial mimicry constitutes a low-level motor process. As shown in Figure 5 the Orbicularis Oculi muscle was significantly more contracted in response to stimuli containing 100% of AU6 (Duchenne’s marker), compared to stimuli with 0% of AU6. More precisely (see Table 3), its contraction was highest in response to “Strongest” smiles, which also received the overall highest judgment of authenticity, and “Weakest With AU6” smiles, which received lower ratings of authenticity. These findings provide preliminary evidence for the embodiment view of facial mimicry as a low-level motor process. Future studies should however test in a more formal way, by recording the activity of several facial muscles at once, whether congruent reactions to facial expressions constitute motor mimicry (the embodiment view), or if they reflect the valence of an internal state.

Findings also showed that facial mimicry influenced participants’ perception of the stimuli, such that the more participants smiled, the higher their ratings of smile authenticity (Figure 2, path b). However, mediation analyses, which asked if participants’ own smiling predicted the effect of smiling intensity in the stimulus on rated authenticity, were not conclusive. Thus, although smiling in participants predicted authenticity ratings, it did not mediate the effect of smiling in the stimulus. This null finding may be explained by the fact that the present measures of smile intensity were not able to fully capture the small changes in activation of the AUs modulated in the stimuli, and of the muscles in the participants’ faces, as well as their interplay. An example of a more fine-grained analysis of facial mimicry, which may have greater potential for resulting in a significant mediation effect, is one that includes trial-by-trial and time-by-time correlations between the stimulus and the participant’s face. A more extensive, and bilateral monitoring of participants’ facial movements (one that is not restricted to 4 muscles) may also be beneficial. Future studies should incorporate these measures.

Nevertheless, the hypothesis that facial mimicry mediates the effect of smile characteristics on rated authenticity remains the most parsimonious one based on the fact that 1) facial mimicry is a costly behavior for the organism, 2) participants spontaneously mimicked the perceived smiles, and 3) this mimicry predicted ratings of authenticity. Importantly, the reverse hypothesis, i.e. that perceived authenticity may have caused participants’ facial reactions, seems less likely based on the finding that participants’ Orbicularis Oculi muscle was most activated in response to two types of smiles that contained the highest degree of the corresponding AU6, but resulted in very different ratings of authenticity.

### Limitations and Future Directions

A number of aspects of both features of the study and its results require further discussion.

First, we chose to construct avatars using a FACS-based platform because they provide an unmatched amount of control over all of the face’s AUs, including the level of activation, symmetry, and dynamics. It could be argued however that avatars themselves are too artificial to provide insight into the present research questions, or that the stimulus construction method yields smiles that lie outside of everyday human expressions. Several arguments speak in favor of the view that despite their artificiality avatar faces are a useful tool to explore the perception, recognition, judgment, and facial mimicry of facial expressions, which are all similar to those elicited during human-to-human interaction.

Humans are becoming more and more accustomed to interacting with human-resembling avatars, because of frequent exposure to animated movies and computer games. Especially individuals of the age of the population used in the present research, who have had significant exposure to computer games and other virtual realities, are able to recognize most emotional expressions in avatar faces just as well as in human faces [77], [78]. An increasing number of studies in the fields of emotion psychology and affective neurosciences use avatar instead of human faces [79]. Furthermore, facial mimicry has been shown to occur to different types of avatars [80]–[87]. And blocking of facial mimicry in response to avatar faces modifies the perception of their facial expressions [4]. Similar to human-to-human interactions, it has been shown that humans prefer to interact with avatars that mimic their behavior, rather than with those who don’t [88]. Finally, ratings of perceived realism were strongly correlated with ratings of authenticity, suggesting that avatars’ smiles were overall perceived as both authentic and likely to occur in human faces.

A potential weakness of the use of avatar faces is the fact that they do not possess feelings or intentions, which casts doubts upon the possibility to judge the authenticity of their smiles. However, research suggests that observers quite easily project human motivations on avatars and robots, and in fact “form relationships with just about anything — regardless of what it looks like” [89]. Moreover, a similar critique may be applied to several of the human faces, which have been used in previous experiments of the same sort. Concretely, much of previous research has employed photos or videos of actors, who were not actually feeling the emotions they portrayed, or at best were feeling them to a lesser extent than their expression suggested [71]. Nevertheless, the significance of our findings should be confirmed using human faces as stimuli instead of avatars.

A second limitation of the stimulus set used here is that the rise time of all AUs was kept constant to 1 second. Certainly, a much greater number of smiles (including asymmetries in onset and peak time, but also in overall amplitude) can be found outside of the laboratory. While we focused on a limited but arguably crucial set of features and kept timing and symmetry constant, others have shown the additional importance of differences in the onset, peak, and offset duration of smiles [11]. In order to reduce the number of factors in the stimuli, with the scope of increasing trial repetition and thus the chance to capture a reliable EMG signal, we opted for limiting the number of smiles to 19. This number, which might seem small compared to the potentially infinite number of combinations of amplitude and timing of bilateral AUs, is nevertheless much bigger than the number of stimuli typically used in facial mimicry studies. Importantly however, all stimuli used in this experiment were created under the supervision of a certified FACS coder, and informally pretested, in order to assure that they appeared ecologically valid.

A third concern arises from our finding that the activity occurring in the lower-face region of the stimuli most strongly determined individuals’ ratings of authenticity. Specifically, analysis of the behavioral data revealed main effects of Zygomaticus contraction and extent of Mouth opening, but no main effect of the presence of crow’s feet, which was the only upper-face feature included (Corrugator activation only occurred in a few trials and was analyzed in a separate analysis). Therefore, it is possible that participants developed a strategy of spending more time looking at the mouth region, knowing that most changes would occur there, compared to the smaller proportion of trials including changes in the eye-region. In doing so, participants may have paid less attention to the changes occurring in the upper face.

Future studies might add eye-tracking measures to control for participants’ fixation of the various subparts of the face, and test judgments of authenticity on a stimulus set including the same number of trials involving movements of the lower and upper face. Still, we remain confident in our facial mimicry results because participants were found to mimic facial movements of the Corrugator and Orbicularis Oculi muscles, both in the upper face, in addition to the Zygomaticus muscle in the lower face.

Another limitation to consider is the fact that we only recorded EMG from the left side of the face, because it is thought to be more expressive than the right side ([90],but see [91]), and it is common practice to do so. However, recent research suggests that timing features of expressions may vary between the right and left side of the face depending on whether the expression is posed or spontaneous [92]. Therefore, it would be interesting in future studies to record the facial EMG from both sides of the face, with the possibility that smiles perceived as more authentic would have an earlier onset in the stimulus’ left side of the face.

Finally, differences in the reliance upon the Duchenne marker for judging smile authenticity might exist across age [31], [93], [94] and cultures [95], implying that depending on the characteristics of the participant pool, slightly different judgments of smile authenticity may be obtained. Differences may also be expected based on participants’ gender, although we were not able to obtain conclusive findings on that issue when excluding male participants from analyses.

To summarize, this research reveals the complexity of the process through which people infer authenticity in dynamic smiles, which is based on the visual perception of all components of smiles, and partly on the facial feedback generated by the observer’s own facial mimicry. Overall, the findings speak in favor of an embodiment view, in which facial mimicry is a low-level motor process contributing to the perception of, and judgment about, facial expressions. However, a mediational role for facial mimicry was not shown. Conclusive evidence may require more fine-grained measures of facial mimicry.
